# Pulcherriminic acid as an antibiofilm effector: expanding *Bacillus* functionality against yeast biofilms

**DOI:** 10.1128/jb.00184-26

**Published:** 2026-08-06

**Authors:** Ramya Srinivasan, Tamil Selvam Saravanan, Soham Utpat, Moshe Shemesh, Satish Kumar Rajasekharan

**Affiliations:** 1 Department of Biotechnology, School of Bioengineering, SRM Institute of Science and Technology 93104, Kattankulathur, Tamil Nadu, India; 2 Department of Food Science, Institute of Postharvest Technology and Food Sciences, Agricultural Research Organization (ARO) 42718, Rishon LeZion, Israel; Dartmouth College Geisel School of Medicine, Hanover, New Hampshire, USA

**Keywords:** pulcherriminic acid, antibiofilm agents, Biofilm dependent modulation, *C. albicans*, biofilm formation, antagonistic*Bacillus*, *B. subtilis*

## Abstract

Pathogenic and persistent biofilms are a major challenge in medicine and industry, acting as resilient microbial communities that resist antibiotics and cause persistent infections. In this review, we discuss pulcherriminic acid, a promising iron-chelating molecule produced by antagonistic *Bacillus subtilis* as a natural weapon against persistent biofilms, particularly those formed by pathogens such as *Candida albicans*. Drawing on recent research, we explore pulcherriminic acid biosynthesis, its native mechanism of depriving fungi of essential iron to disrupt their growth and virulence, and innovative methods to enhance its production through metabolic engineering. We also highlight its potential applications, from fighting infections to serving as a biocontrol agent in agriculture and even as a natural preservative in food. By harnessing this *Bacillus*-derived metabolite, we suggest sustainable ideas for tackling antibiotic resistance and persistent biofilm-related problems.

## MULTIFACETED ROLES OF IRON IN BIOFILM DEVELOPMENT

Iron is a vital element of biofilm-associated pathogenicity because it fuels core metabolic functions, including respiration, DNA synthesis, and redox reactions that support microbial growth and stress tolerance in structured communities (1, 2). In many bacterial pathogens, iron availability acts as a key environmental cue that regulates the transition from planktonic to biofilm lifestyle by modulating siderophore production, iron-uptake systems, and global regulatory networks controlling adhesins, extracellular polymeric substances, and matrix architecture (3–5). Low iron generally triggers aggressive iron-scavenging and biofilm-promoting responses, enhancing persistence in hostile host niches, whereas excess iron can also promote biofilm development through oxidative stress-driven increases in extracellular DNA and polysaccharides, as seen in *Campylobacter jejuni* and other food-borne or nosocomial pathogens (3, 6, 7). This tight interdependence between iron homeostasis and biofilm formation not only explains why iron-rich microenvironments often correlate with chronic, recalcitrant infections but also underpins emerging anti-biofilm strategies based on iron chelation, siderophore hijacking, or disruption of intracellular iron mobilization to weaken biofilm stability and resensitize pathogens to antibiotics (2, 3).

The *Bacillus* species, such as *B. subtilis* and *B. velezensis,* are an important source of diverse secondary metabolites with vast antimicrobial potential (8–10). Approximately 4%–5% of the *B. subtilis* genome is dedicated to the biosynthesis of antimicrobial compounds, including lipopeptides such as surfactin, fengycin, and iturin (8, 10). These metabolites, along with bacteriocins and extracellular lytic enzymes, contribute to the biocontrol activity of *Bacillus* species by inhibiting pathogen growth and biofilm formation by interfering with quorum sensing pathways (11). In addition, certain *Bacillus* lipopeptides exhibit anti-adhesive effects that may reduce the initial attachment of pathogenic microorganisms to surfaces, thereby limiting early-stage biofilm development (12).

Central to this antagonistic capability is the production of pulcherriminic acid, a cyclic dipeptide synthesized via the cyclization of two leucyl-tRNA molecules by the cyclodipeptide synthase *YvmC* and subsequent oxidation by the cytochrome P450 oxidase *CypX* (13, 14). Once secreted, pulcherriminic acid functions as a high-affinity iron chelator that non-enzymatically binds extracellular ferric iron Fe^3+^ to form reddish-brown pigment known as pulcherrimin (15, 16). By sequestering environmental iron into pulcherrimin, it can be hypothesized that the *Bacillus* cells effectively deplete the free iron, which leads to compromised growth through limiting the iron availability to the competing microbial biofilms.

## DISCOVERY OF PULCHERRIMINIC ACID

**Fig 1:**
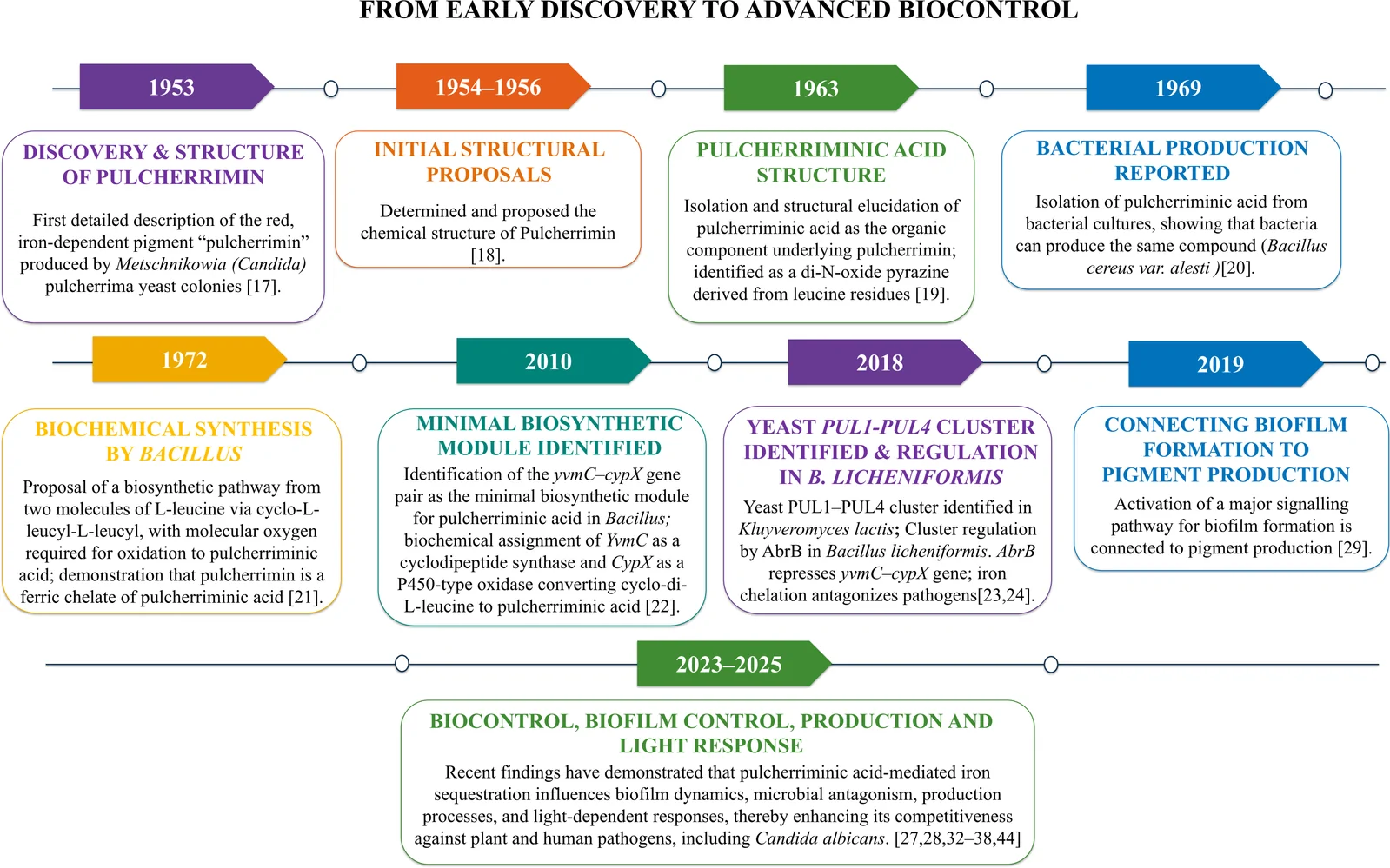
Timeline of the pulcherrimin and pulcherriminic acid research from its early discovery to the advanced biocontrol agent.

Pulcherriminic acid, a cyclic dipeptide with strong iron-chelating potential, was first described in 1953 by Dutch microbiologist A.J. Kluyver and his team. Isolated from the yeast *Metschnikowia pulcherrima* (previously *Candida pulcherrima*) as the precursor to the red pigment pulcherrimin, the compound was initially noted for its critical role in binding iron during microbial growth (17). Early structural studies in the mid-1950s by Cook and Slater proposed tentative piperazine-based formulas based on the isolation from yeast cultures; however, inconsistencies in optical activity and hydrogenation products prompted further refinement (18). In 1963, J.C. MacDonald finally elucidated its structure as 2,5-diisobutyl-3,6-dihydroxy-pyrazine-1,4-dioxide through NMR spectroscopy, elemental analysis, and reduction to DL-leucine anhydride from multiple *C. pulcherrima* strains, establishing it as a key microbial secondary metabolite (19). Research shifted to biosynthetic pathways in the 1970s, with Robert L. Uffen and E. Canale-Parola demonstrated in 1972 that *B. subtilis* synthesizes pulcherriminic acid from two L-leucine molecules via the intermediate cyclodileucine (cyclo-L-Leu-L-Leu), as confirmed through radiolabeling experiments in leucine auxotrophs (20, 21). By 2010, the bacterial gene cluster (*yvmC* encoding cyclo-di-leucine synthase and *cypX* for oxidation) was identified in *B. subtilis* and *B. licheniformis*, detailing the multi-step oxidation process (22). In yeasts, the *PUL1-PUL4* cluster was first characterized in *Kluyveromyces lactis* in 2018 by Krause et al., with *PUL1* and *PUL2* as core biosynthetic genes, followed by confirmation in *M. pulcherrima*, revealing convergent evolution despite identical products (23; Fig. 1).

## RECENT FOCUS ON *BACILL*US-DERIVED PULCHERRIMINIC ACID

Recent research on *Bacillus*-derived pulcherriminic acid has surged since 2013, bridging a decade-long gap following its initial identification in 1972 in *B. subtilis*, driven by advances in genomics, transcriptomics, and biofilm biology that have unveiled its multifaceted roles beyond iron chelation (21, 24, 25). Advancements from the 2020s till now have illuminated the intricate regulation of pulcherriminic acid biosynthesis in *B. subtilis*, where transcription factors AbrB, ScoC, and the MarR-family PchR orchestrate temporal expression of the *yvmC-cypX* cluster during exponential-to-stationary phase transitions, optimizing iron chelation for biofilm maturation (14, 26). Beyond simple sequestration, pulcherriminic acid serves as a protective antioxidant against oxidative stress and acts as an intercellular signal that modulates the transition from exponential growth to the stationary phase within the biofilm community (13, 16). Additionally, pulcherriminic acid mediates light-dependent biofilm responses, enhancing photoprotection and community resilience under environmental fluctuations (27).

## PULCHERRIMIN TO HINDER YEAST BIOFILM PATHWAYS

**Fig 2:**
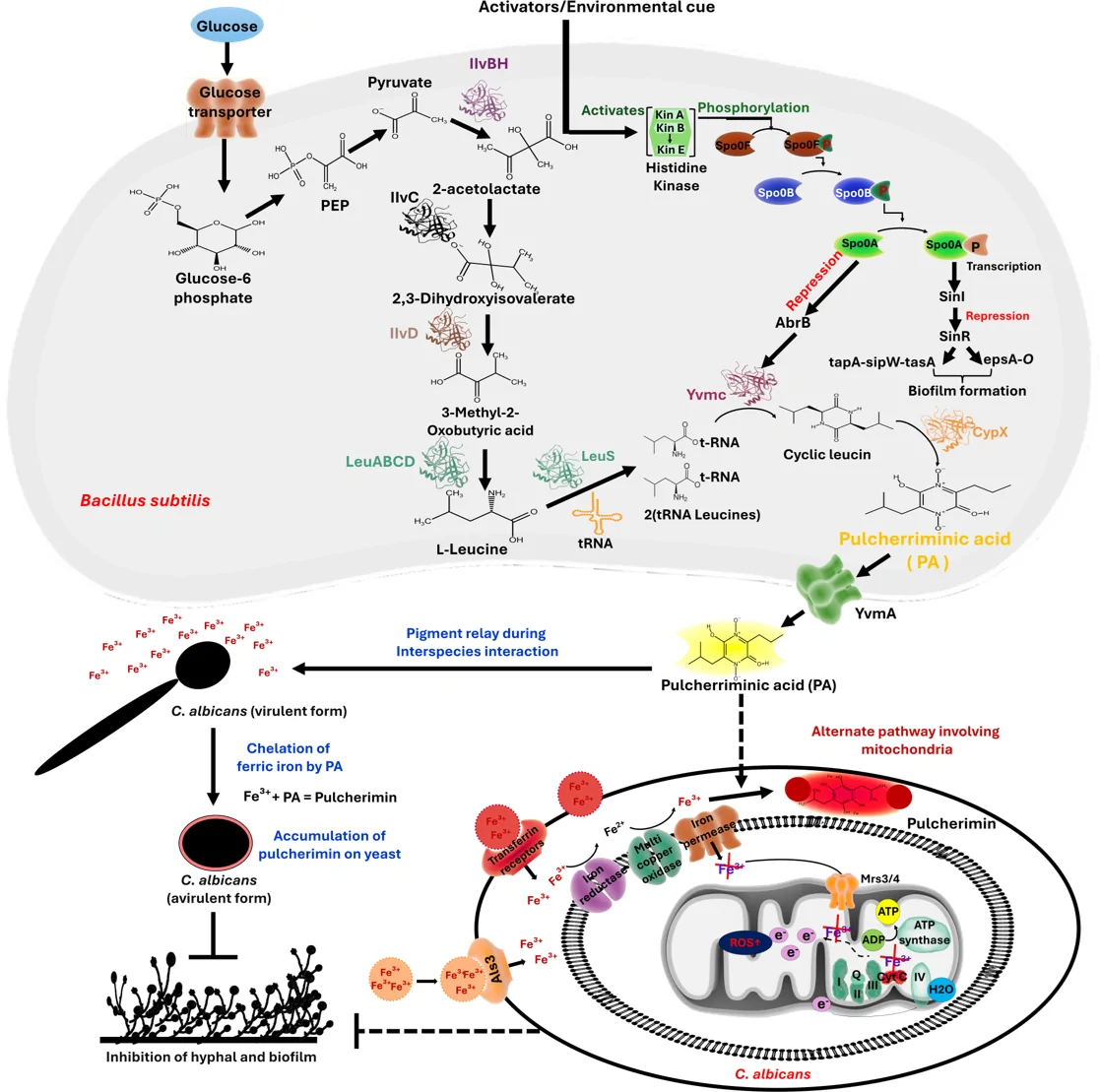
Iron sequestration by *B. subtilis* and its role in disrupting *C. albicans* biofilm through pulcherriminic acid production from glucose. Pulcherriminic acid is secreted out of the cell, which forms a reddish-brown pigment - pulcherrimin in contact with Fe^3+^ ions. Overall, the diagram depicts the regulatory pathways involving pulcherrimin biosynthesis and biofilm formation in *B. subtilis,* which further plays a vital role in antagonistic interactions with *C. albicans*.

Pulcherriminic acid-mediated antagonism is not limited to a simple pigment-forming reaction but is closely connected to microbial iron homeostasis and iron-dependent physiology (16, 28). In *B. subtilis*, pulcherrimin biosynthesis begins with glucose-derived carbon flux entering the leucine biosynthetic pathway, leading to the formation of L-leucine. L-Leucine is activated as leucyl-tRNA by *LeuS*, and two leucyl-tRNA molecules are condensed by the cyclodipeptide synthase *YvmC* to form cyclo-L-leucyl-L-leucyl (26, 29, 30). This cyclic dipeptide is then oxidized by the cytochrome P450 enzyme *CypX* to produce pulcherriminic acid, which is relayed outside the cell and chelates Fe³^+^ to form the insoluble reddish pigment pulcherrimin (22, 26). This pathway is also linked to biofilm development, where environmental cues activate the Kin–Spo0A phosphorylation cascade, relieving *SinR*-mediated repression through *SinI* and supporting matrix-associated biofilm formation that helps localize pulcherrimin production (14, 16, 28; Fig. 2).

Pulcherriminic acid-producing microorganisms, including *B. subtilis*, *B. licheniformis*, and *M. pulcherrima,* exploit iron sequestration mechanisms for broad-spectrum biocontrol, targeting diverse fungal and bacterial pathogens mostly through nutritional competition, restriction of bioavailable iron rather than direct toxicity. Notably, pulcherriminic acid-producing strains suppress phytopathogens such as *Botrytis cinerea* (gray mold), *Fusarium oxysporum* (vascular wilt), and *Pseudomonas syringae* pv. *tomato* DC3000 (24, 31–35). In these cases, iron immobilization by *Metschnikowia spp*. reduces spore germination and mycelial proliferation and disrupts fungal growth (24, 32, 33). Recent findings further extend this antagonistic concept to human pathogens, revealing effective inhibition of *C. albicans* biofilms via localized iron chelation. For instance, pulcherriminic acid acts as a multifunctional molecule with strong anti-*Candida* potential (28). Within biofilms, *B. subtilis* employs a “relay” mechanism, actively transporting pulcherriminic acid to zones of fungal colonization. This targeted delivery ensures precise iron sequestration at infection interfaces, thereby arresting *C. albicans* expansion and preventing morphological transition from yeast to hyphal forms. Mechanistically, pulcherriminic acid disrupts *C. albicans* iron uptake networks, inducing compensatory activation of high-affinity systems such as reductive iron assimilation and siderophore-mediated transport (36). However, the strong chelation capacity of pulcherrimin often overwhelms these responses, sustaining an iron-starved state that downregulates hypha-specific and virulence-associated genes. Thus, pulcherriminic acid functions as both an ecological defense metabolite and a promising therapeutic lead, targeting iron-dependent virulence and biofilm formation through a precisely localized, relay-based mechanism.

## IRON SEQUESTRATION AND ALTERNATIVE PATHWAYS

Alternatively, we propose the possibility of other pathways by which pulcherriminic acid, produced by *B. subtilis,* could exert its effect on *C. albicans* primarily through iron sequestration. *C. albicans* is known to depend on iron for vital cellular processes, particularly mitochondrial functions such as iron-sulfur cluster (ISC) biosynthesis and electron transport chain (ETC) activity (37). In fungi, iron is transported into the mitochondria via the Mrs3 and Mrs4 transporters, where it may be incorporated into ISC with the help of chaperones like Mge1 (38, 39). These clusters are believed to be essential for several mitochondrial proteins, including those involved in electron transport and energy production. Treatment with pulcherriminic acid could significantly alter the ETC activity; for instance, components such as cytochrome c, succinate dehydrogenase, and cytochrome c oxidase I are likely to rely on iron for their function. Iron limitation due to the formation of pulcherriminic acid-iron complex results in the malfunctioning of the ETC. As reported earlier, electrons might prematurely escape from the ETC and react with oxygen, forming reactive oxygen species (ROS) (40). The accumulation of ROS may induce oxidative stress, potentially causing damage to cellular components such as proteins, lipids, and DNA. This oxidative imbalance could weaken the fungal ability to proliferate or survive under iron-restricted conditions.

Beyond metabolic disruption, the iron-limiting environment created by the pulcherrimin formation also impairs *C. albicans* biofilm. It is likely that, without sufficient iron, fungal cells struggle to maintain essential functions, thereby inhibiting biofilm formation and reducing survival (Fig. 2).

## GROWING INTEREST IN BIOPROSPECTING OF PULCHERRIMINIC ACID FOR MEDICAL USE

**Fig 3:**
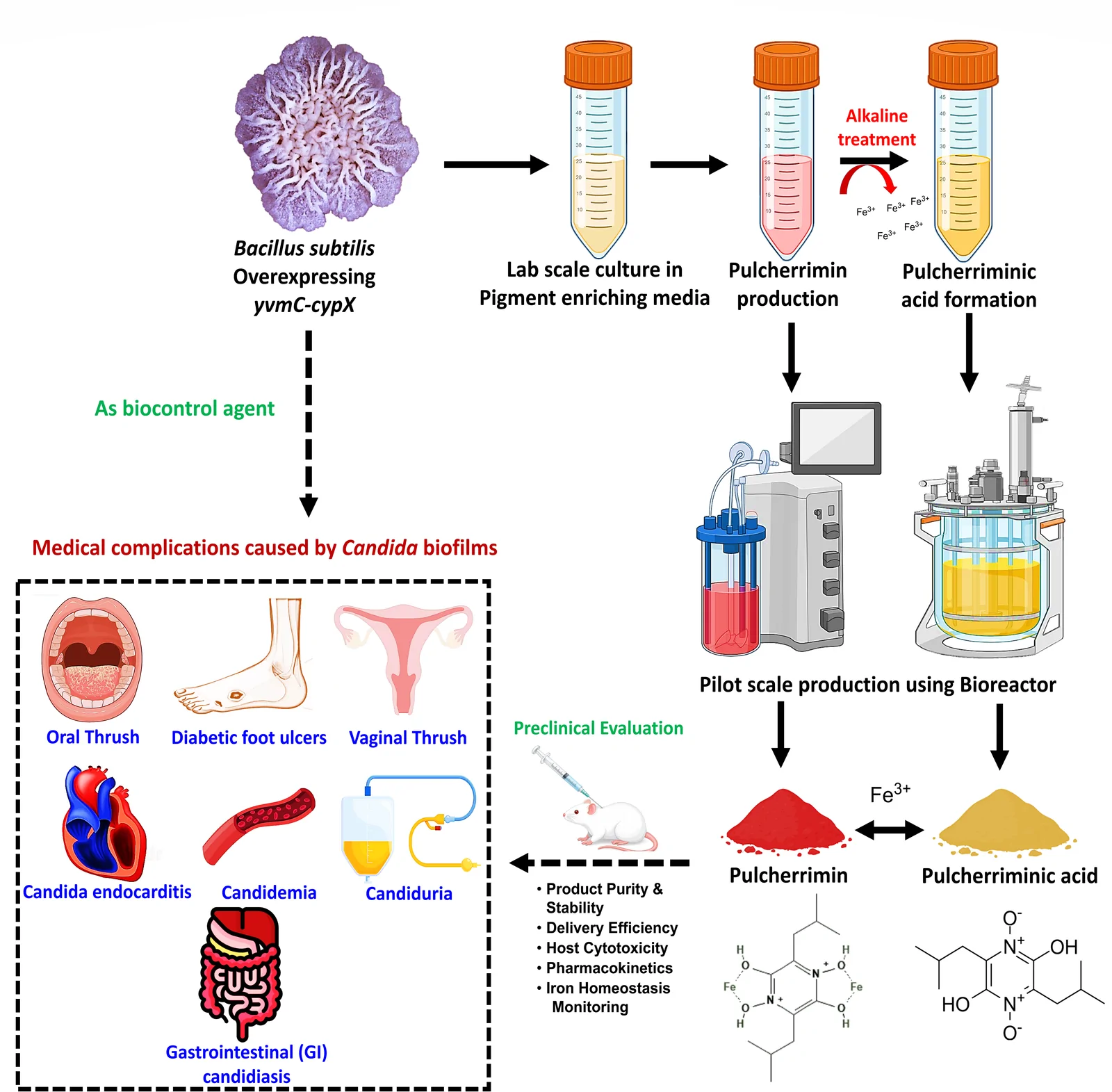
Schematic representation of pulcherrimin and pulcherriminic acid biosynthesis and its multifarious applications. *B. subtilis* overexpressing the *yvmC–cypX* operon grown in lab or pilot scales for its upscaling production. The molecules show multifaceted applications for the growth and welfare of green, white, and red biotechnology.

Recent advances in microbial engineering have renewed interest in the bioprospecting of pulcherriminic acid for biomedical and biotechnological applications. Chen et al. (41) highlighted that *B. subtilis* PS832 can be optimized as a sustainable microbial cell factory for pulcherrimin production by relieving transcriptional repression in the *yvmC–cypX* operon, responsible for biosynthesis of pulcherriminic acid prior to converting it into pulcherrimin (41). As depicted in Fig. 3, lab-scale production will enable enhanced pulcherrimin accumulation, while alkaline treatment will facilitate its conversion into pulcherriminic acid, thereby supporting downstream scale-up through pilot-level bioreactor production. The Fe³⁺-dependent interconversion between pulcherriminic acid and pulcherrimin will further underscore iron chelation as the central biochemical basis of its antimicrobial and antifungal activity (31–34). Importantly, this system will extend beyond industrial biomanufacturing by demonstrating potential biocontrol applications against clinically significant *Candida*-associated complications, including mucosal, gastrointestinal, catheter-associated, bloodstream, and device-related infections. Collectively, these advances will reinforce *B. subtilis* not merely as a classical model organism but as a programmable cell factory for naturally derived bioactive metabolites with translational value in biotechnology, agriculture, and biomedical infection control.

While pulcherriminic acid exhibits considerable potential as an anti-biofilm agent, its translational applicability remains largely unexplored. Therefore, systematic investigations addressing its stability, biocompatibility, delivery strategies, pharmacokinetics, and effects on host iron homeostasis are warranted (Fig. 3). Such studies will be essential to establish its feasibility for biomedical applications, particularly in localized anti-biofilm interventions. Overall, this mini-review is believed to provide a comprehensive perspective on the current state of knowledge and stimulate further research into the therapeutic and biotechnological potential of pulcherriminic acid.
